# Virtual Reality Rehabilitation Systems for Cancer Survivors: A Narrative Review of the Literature

**DOI:** 10.3390/cancers14133163

**Published:** 2022-06-28

**Authors:** Antonio Melillo, Andrea Chirico, Giuseppe De Pietro, Luigi Gallo, Giuseppe Caggianese, Daniela Barone, Michelino De Laurentiis, Antonio Giordano

**Affiliations:** 1 Department of Mental and Physical Health and Preventive Medicine, “Luigi Vanvitelli” University of Campania, 80129 Naples, Italy; antonio.melillo2@studenti.unicampania.it; 2 Department of Biology, Sbarro Institute for Cancer Research and Molecular Medicine, Center for Biotechnology, College of Science and Technology, Temple University, Philadelphia, PA 19122, USA; giordano@temple.edu; 3 Department of Social and Developmental Psychology, “Sapienza” University of Rome, 00185 Rome, Italy; andrea.chirico@uniroma1.it; 4 Institute for High Performance Computing and Networking, National Research Council of Italy (ICAR-CNR), 80131 Naples, Italy; giuseppe.depietro@icar.cnr.it (G.D.P.); luigi.gallo@icar.cnr.it (L.G.); giuseppe.caggianese@icar.cnr.it (G.C.); 5 Cell Biology and Biotherapy Unit, Istituto Nazionale Tumori-IRCCS-Fondazione G. Pascale, 80131 Naples, Italy; d.barone@istitutotumori.na.it; 6 Department of Breast and Thoracic Oncology, Istituto Nazionale Tumori-IRCCS-Fondazione G. Pascale, 80131 Napoli, Italia

**Keywords:** virtual, reality, cancer, rehabilitation, disability, robotics, lymphedema, pain, fatigue, telemedicine

## Abstract

**Simple Summary:**

To the best of our knowledge, this is the first review aiming to assess the impact of VR on the rehabilitation care of cancer survivors. We conducted a general review of the current evidence on the efficacy of virtual reality rehabilitation (VRR) systems on cancer-related impairments as retrieved through a systematic search of the main research databases. VRR systems may improve adherence to rehabilitation training programs and be better tailored to cancer patients’ needs, but more data is needed.

**Abstract:**

Rehabilitation plays a crucial role in cancer care, as the functioning of cancer survivors is frequently compromised by impairments that can result from the disease itself but also from the long-term sequelae of the treatment. Nevertheless, the current literature shows that only a minority of patients receive physical and/or cognitive rehabilitation. This lack of rehabilitative care is a consequence of many factors, one of which includes the transportation issues linked to disability that limit the patient’s access to rehabilitation facilities. The recent COVID-19 pandemic has further shown the benefits of improving telemedicine and home-based rehabilitative interventions to facilitate the delivery of rehabilitation programs when attendance at healthcare facilities is an obstacle. In recent years, researchers have been investigating the benefits of the application of virtual reality to rehabilitation. Virtual reality is shown to improve adherence and training intensity through gamification, allow the replication of real-life scenarios, and stimulate patients in a multimodal manner. In our present work, we offer an overview of the present literature on virtual reality-implemented cancer rehabilitation. The existence of wide margins for technological development allows us to expect further improvements, but more randomized controlled trials are needed to confirm the hypothesis that VRR may improve adherence rates and facilitate telerehabilitation.

## 1. Introduction

Cancer ranks as a leading healthcare issue, striking 19.3 million new cases worldwide in just 2020 and with an estimated projection of 28.4 million new cases for 2040 [1]. Contemporarily to this increase in incidence, mainly explainable by the world population’s growth and aging, cancer mortality rates have been steadily decreasing by 1% per year, both in high- and low-income countries and for both sexes [2]. Thanks to both diagnostic and therapeutic advancements, the 5-year survival rate of cancer patients has indeed increased from 49% in 1979 to roughly 67% in the US in 2015 [3,4]. As a consequence of these trends, the population of individuals who have received a cancer diagnosis in their life is set to increase rapidly, with the latest projections showing an increase from 16.9 million in the US to 26.1 million people in 2040 [5]. “Cancer survivors” is a term generally used to define anyone living with the physical and or psychological consequences of a recent or past cancer diagnosis and its treatment, with some researchers even advocating for the inclusion of even cancer patients’ caregivers and family members under the term [6]. These consequences have a long and significant impact on the physical functioning of this population, as both the disease, the long-term toxicity of chemotherapeutic drugs and radiotherapy, as well as surgical procedures can result in chronic symptoms and long-standing physical and cognitive impairment.

Pain is by far one of the most common chronic symptoms cancer survivors experience, with prevalence rates of 55.0% during anticancer treatment, 39.3% after curative treatment, and 66.4% in advanced, metastatic, or terminal disease [7]. Persistent pain not only significantly undermines quality of life but also causes functional limitations and hence disability. Cancer-related fatigue (CRF) is another extremely common symptom in cancer patients, with a prevalence ranging from 25% to 99% depending on the specific disease, the treatment, and age [8]. Lymphedema is an extremely frequent consequence of cancer treatment, as it can be secondary to the surgical removal of lymph nodes, radiation therapy, chemotherapy, or a combination of such [9]. The condition may severely impact patients’ lives, as it causes both pain and function limitations. Its incidence is influenced by both the cancer and the intervention type: rates range from 75% of breast cancer patients after axillary nodes removal to between 14.5 and 41.4% after chest and breast radiation therapy depending on the extension of the area involved, to 50% for melanoma patients and a 16% incidence for genitourinary cancers [10,11]. Many cancer survivors experience not only physical but also cognitive impairment, in particular in areas such as memory, attention span, word-finding, and speed of processing and execution. This impairment is sometimes colloquially referred to as “chemo brain”, referring to the well-known neurotoxicity of many chemotherapeutic drugs [12]. However, recent findings on the existence of mild cognitive impairment already existing before chemo treatment pose doubts on the true cause(s) of this condition [13]. Chemotherapy-induced peripheral neuropathy (CIPN) is a severe collateral effect of chemotherapy. Many chemotherapeutic drugs can indeed cause different types of nerve damage depending on the exact chemical compound [14]. Its incidence also varies depending on the treatment, ranging from 19% to 85%. Clinically, CIPN usually manifests itself mainly as a distal sensory deficit, with symptoms of dysesthesia, paresthesia, pain symptoms, or complete anesthesia. Motor symptoms occur less frequently and also usually involve distal limbs, causing balance and gait problems as well. CIPN usually gradually develops months after chemotherapeutic treatment and may affect the patient for years.

These conditions have been shown to benefit from rehabilitation, and in the last years, many systematic reviews and guidelines have contributed to the establishment of specific recommendations for the prescription of specific exercise programs for different cancer types [15,16,17,18,19]. Despite this indication, many studies have shown how just a minority of cancer survivors are referred to rehabilitation programs. Reporting data collected from 163 breast cancer survivors, Cheville et al. found that 91% of women had physical impairments, but only 30% were receiving proper rehabilitative care [20]. Concordantly, a study by Hansen et al. examining a cohort of 3439 cancer survivors reported a total of 60% of patients referring to the unmet need for either physical or psychological rehabilitation [21]. In a more recent 20-year follow-up of pediatric brain cancer survivors in Norway, the percentage rose to as high as 86% [22]. Through a non-systematic review of the previous literature, Cheville attempted to explain the lack of proper rehabilitative care, mentioning as possible causes the insidious and gradual genesis of these impairments as well as the incapability of the cancer care system to deliver the early detection of the impairing symptoms [23]. However, even when the program is initiated, it is often discontinued as early as within the first twelve months, mainly as a result of the difficulty of traditional training programs in motivating the patients’ adherence [24]. In addition, the recent pandemic has very well exposed another cause of this underutilization of rehabilitative cancer care, which is the inadequacy of the present rehabilitative care system in delivering home-based interventions [25,26]. Indeed, many cancer survivors suffer from disabilities or transportation issues which may limit their attendance at rehabilitation facilities. Therefore, in the last years, many studies have been investigating the role of telerehabilitation in the rehabilitative care of cancer survivors to improve adherence and as a safe and more accessible alternative to traditional rehabilitation [27,28,29]. One of the latest technologies proposed to remotely connect patients and rehabilitation professionals is Virtual Reality (VR) [26,30,31,32,33,34]. Virtual Reality Rehabilitation (VRR) has been tested in various clinical conditions, such as stroke-related deficits [35], spinal cord injuries [36], multiple sclerosis [37], Parkinson’s disease [32], cerebral palsy [38,39,40], and cancer rehabilitation. Many studies have argued that VRR may improve both adherence rates and training intensity thanks to its entertaining and game-like nature [41,42,43].

The purpose of the present narrative review is to contribute to the investigation of whether VR may be a useful implementation in the cancer rehabilitation field and to give an overview of the current evidence on this application. At the moment, the scientific literature registers either attempts to evaluate the advantages of VR implementation in the rehabilitation field in general [41,44] or to review the implementation of VR in palliative care for single cancer symptoms, mainly during acute cancer care, as highlighted by Zeng et al. [45,46]. From our perspective, the former fails to assess the advantages of VR-integrated rehabilitation when applied to the specifics of cancer survivor disabilities, which often result from the slow and insidious accrual of more symptoms and physical impairments [20]. The latter, on the other hand, does not examine the potential application of VR technology to cancer survivors with chronic symptoms and their role in an impairment-driven rehabilitation of disabilities resulting from a cancer history. Hence, to the best of our knowledge, this is the first review aiming to assess the impact of VR on the rehabilitation care of cancer survivors.

## 2. Methods

### Database Search

The main online databases (PubMed, Scopus) were searched from inception until May 2022. The query string was the following: Cancer Survivor*” OR “cancer” OR “cancer patient*” AND “Lymphedema” OR “cancer-related fatigue” OR “Fatigue” OR “Chronic Pain” OR “Cancer Pain” OR “cognitive” OR “motor” OR “symptom management” OR “peripheral neuropathy” AND “Rehabilitation” OR “Telerehabilitation” OR “Exercise” OR “physical therapy” OR “sensorimotor rehabilitation” OR “exercise training” OR “postural balance” OR “sensorimotor” AND “Virtual Reality” OR “body sensors” OR “avatar*”. The first author performed the literature search. The first and second authors independently screened titles and abstracts as well as full texts’ reference lists against eligibility criteria. The final selection of articles was discussed by the first and second authors. Study eligibility was assessed using the PICOS tool [47]: to be included, studies had to fulfill the following inclusion criteria: (1) population: individuals with a history of cancer; (2) intervention: Virtual Reality-based rehabilitation; (3) comparison for RCCTs: standard physiotherapy; (4) outcomes for clinical trials: functional parameters, pain, lymphedema volume, cancer-related fatigue, program adherence, exercise performance; and (5) study design: RCT with or without control, perspective studies, comparative studies, feasibility studies. Studies published in English, Spanish, or Italian were all considered.

## 3. Results

**Figure 1:**
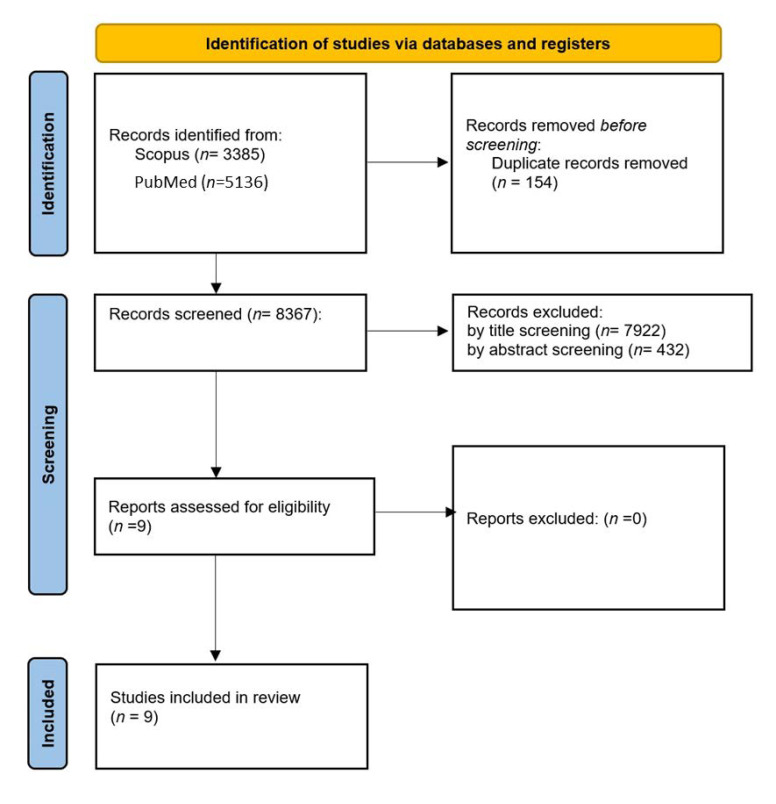
Prisma flowchart of the study selection.

The search of the main databases (PubMed, Scopus) produced a total of 7733 results. Duplicate detection led to the elimination of 149 results. After screening through eligibility criteria, a total of nine studies were selected for our review (Figure 1). We will here, therefore, review the design of the included studies, summarized in Table 1.

Atef et al. conducted a quasi-randomized clinical trial comparing the efficacy of VRR and proprioceptive neuromuscular facilitation (PNF) on post-mastectomy lymphedema upper-arm exceeding volume and upper arm function recovery, measured through the QuickDASH-9 scale [48]. The experimental procedure consisted of a 30 min exercise program using a Wii Fit non-immersive VR game. Both the VRR and the PNF procedures were conducted two times per week for a total of 4 weeks. During these sessions, both groups, consisting of 15 women each, also received a procedure of pneumatic compression for the treatment of lymphedema.

Axenie and Kurz conducted a prospective study on the combination of Virtual Reality avatars and Machine Learning to drive patient-tailored CIPN-related motor deficit compensation [49]. They proposed a closed-loop system based on wearable devices designed to precisely assess the kinematics of the sensorimotor deficits. Furthermore, they conceptualized a VR avatar designed to reproduce the patient’s movements and to display the discrepancies between the desired movement and the measured/executed one, so as to trigger deficit compensation.

Basha et al. conducted a randomized clinical trial comparing the therapeutic efficiency of non-immersive VR training and resistance exercise training on breast cancer-related lymphedema [50]. The experimental protocol consisted of an exercise program conducted through Xbox Kinect games involving upper arm motion. Both rehabilitation groups, consisting of 30 patients each, received five rehabilitation sessions per week for 8 weeks. The outcome measures included excessive limb volume and pain, measured through the visual analog scale (VAS); the impairment of the upper arm, measured through the Disability of the Arm, Shoulder, and Hand (DASH) questionnaire; shoulder range of motion (ROM); shoulder muscle strength; and hand grip strength.

Feyzioğlu et al., 2019 presented a prospective randomized controlled trial comparing the efficacy of a non-immersive VRR intervention with standard physiotherapy on breast cancer survivors who had undergone surgery with axillary dissection [51]. The experimental and control groups, both consisting of 20 individuals, both received the treatment for 45 min per session and two times a week for 6 weeks. The experimental intervention consisted of playing Xbox Kinect games involving upper arm motion in the presence of a trained physiotherapist. However, the intervention group also received a scar tissue massage for 5 min and passive shoulder joint mobilization for 5 min, performed by the same physiotherapist assisting them. The outcomes considered were pain (VAS), grip strength, functionality (assessed through the DASH questionnaire), muscle strength, ROM, and fear of movement, measured through the Tampa Kinesiophobia Scale (TKS).

Hoffman et al. (2014) conducted a non-controlled trial investigating the feasibility of a home-based VRR intervention on seven lung cancer patients who had received thoracotomy [52]. The home-based rehabilitation program, divided into two phases of 5 and 10 weeks, respectively, consisted of playing Nintendo Wii Fit Plus exergames of gradually increasing intensity and duration 5 days a week. The VRR sessions did not require the presence of rehabilitation professionals. The outcomes considered were the levels of adherence, measured as the days of actual training, exercise performance, cancer-related fatigue (0–10 scale), perceived self-efficacy for fatigue self-management (0–10 scale), and perceived self-efficacy for walking 30 min (%).

House et al. conducted a trial on a sample of six patients to investigate the feasibility of a rehabilitative intervention based on a novel technology, named BrightArm Duo, on breast cancer survivors with post-surgical pain and depression [53]. The novel technological tool tested consisted of a combination of a robotic table for forearm rehabilitation and a computer executing non-immersive VR rehabilitation games. The rehabilitation program consisted of training sessions lasting 20 to 50 min of training twice a week for a period of 8 weeks. The outcomes considered were pain, measured through the Numeric Rating Scale (NRS); arm, hand, and bimanual function measured through the Fulg-Meyer assessment, the Chedokee arm and hand activity inventory, and the Jebsen hand function test; upper arm autonomy in the activities of daily living, measured through the Upper extremity function index (UEFI-20); depression, measured through the Beck Depression Inventory (BDI-II); and cognitive function, measured through the Neuropsychological Assessment Battery (NAB), the Hopkins Verbal Learning Test (HVLT-R), the Brief Visuospatial Memory Test (BVMT-R), and the Trail Making Test (TMT).

Reynolds et al. conducted a pilot study to evaluate the efficacy of two different VRR interventions on pain, CRF, and quality of life [54]. The study involved two groups of 19 and 20 women with metastatic breast cancer who were asked to participate in an immersive home-based VR intervention. The technology involved consisted of a Pico Goblin VR headset playing two different relaxing scenarios. The outcomes considered were pain, measured through the Brief Pain Inventory scale (BPI); quality of life, measured through the EQ-5D-5L scale; fatigue, measured through the Functional Assessment of Chronic Illness Therapy Fatigue scale (FACIT-Fatigue); and depression, anxiety, and stress levels, measured through the short version of the Depression, Anxiety, and Stress Scales (DASS-SF).

Schwenk and colleagues conducted a randomized trial on VR-based balance training [55]. The authors used inertial sensors equipped with gyroscopes and accelerometers on the lower limbs to assess positions and joint angles and a multi-step balance retraining virtual game based on the inputs of the sensors. In particular, the intervention group, consisting of 11 individuals with chemotherapy-induced polyneuropathy, conducted exercises and balance retraining tasks while receiving visual and auditory feedback on their motor errors. The outcomes measured were the sway of the hip, the sway of the ankle, the center of mass movement, gait speed, and fear of falling, measured through the Falls Efficacy Scale (FES-I).

Tsuda et al. conducted a preliminary study on a VR-based exercise program on over 60-year-old hospitalized patients with hematological malignancies receiving chemotherapy [56]. The virtual reality exercise program involved Nintendo Wii Fit games, which were played for 20 min a day, five times a week until hospital discharge. The primary outcomes were adherence rates, physical performance (measured through the Barthel index), muscle strength, and emotive state (hospital anxiety and depression scale).

In summary, eight of the considered studies were clinical trials, with one study conducting a preclinical investigation [49]. Of the clinical trials, four compared VRR to a standard rehabilitation program [48,50,51,55]. One study involved an immersive VR program [54], while the remaining eight studies used non-immersive VR technology. As for the population considered by the clinical trials, five of the included studies involved breast cancer survivors [48,50,51,53,54]. As for the outcomes considered, four of the retrieved studies tested VRR on more than one physical impairment [50,51,53,54]. Overall, we found four studies testing the efficacy of VRR on chronic pain [50,51,53,54], two studies on cancer fatigue [52,54], two studies on lymphedema-related excessive arm volume [48,50], one on cognitive function [53], four on motor performance impairment [48,50,51,53], and two on chemotherapy-induced polyneuropathy [49,55]. Finally, we here report the results of the two included studies considering adherence rates as an outcome [52,56].

### 3.1. Pain

Feyzioğlu et al. did not find a statistical difference in pain [51]. The study, however, found significant differences in the decreased fear of movement as calculated through the Tampa Kinesiophobia Scale. Moreover, House et al. reported a 20% decrease in pain after treatment (*p* = 0.1) [53]. Basha and colleagues, comparing non-immersive VR exercise with regular resistance exercise in patients with breast cancer-related lymphedema, found significant differences in pain intensity (*p* = 0.002) between groups [50]. Reynolds et al. found that both scenarios significantly reduced pain (mean difference = −6.01, *p* = 0.004) [54]. To summarize, four of the included studies considered pain as their outcome, but only two found a statistically significant effect.

### 3.2. Fatigue

Hoffman et al. reported statistically significant improvements in both CRF severity and perceived self-efficacy for walking [52]. Reynolds et al. found a statistical difference in pain and at follow-up compared to before the intervention (mean difference −5.00, *p* < 0.001) [54]. To summarize, two of the included studies found statistically significant effects of VR on cancer-related fatigue.

### 3.3. Lymphedema

Atef et al. found that both VR and PNF exercise reduced edema, with no significant differences (*p* = 0.902) [48]. Basha et al.’s trial showed no significant differences among groups for lymphedema-related excessive shoulder volume (mean difference = −11.1 mL, *p* = 0.15) [50]. In conclusion, none of the included studies found statistically significant evidence in favor of a VRR intervention compared to standard rehabilitation.

### 3.4. Cognitive Impairment

House et al.’s study on VR rehabilitation found it effective on cognitive function, with 10 out of 11 parameters improved (*p* = 0.004) [53].

### 3.5. Motor Performance

The Feyzioğlu trial on arm rehabilitation following mastectomy recorded improvements in range of motion, grip strength, and arm muscle strength but did not find any significant differences with the control group [51]. House et al.’s study, also considering arm rehabilitation in breast cancer patients following surgery, reported a significant improvement of the affected shoulder in 17 of 18 range-of-motion metrics (*p* < 0.01), of which five were above the Minimal Clinically Important Difference [53]. The study also reported a recovery in 13 out of 15 strength and function metrics (*p* = 0.02). Basha et al.’s trial also found statistical differences in physical and motility outcomes (shoulder flexion strength, external rotation strength, abduction strength, and handgrip strength) in favor of the control group, who performed regular resistance exercises [50]. The trial also reported that VRR was, however, significantly superior to standard rehabilitation for the range of motion outcome (*p* < 0.001). Lastly, the Atef et al. trial reported statistically significant differences among the VRR group and the control group regarding the functional improvements of the arm following mastectomy (*p* = 0.045) [48]. To summarize, four trials considered motor impairment as their outcome, but only two reported a statistically significant effect of VRR, while one trial found it inferior compared to standard rehabilitation on some of the considered outcomes.

### 3.6. Chemotherapy-Induced Peripheral Neuropathy

Schwenk et al. reported how the sway of the hip, ankle, and center of mass while standing with eyes opened and in a semi-tandem position was significantly reduced in the intervention group compared to the control (*p* = 0.010–0.022 and *p* = 0.008–0.035, respectively, for the two positions) [55]. No significant effects were found for balance with eyes closed, gait speed, and fear of falling (*p* > 0.05).

### 3.7. Adherence to Rehabilitation Programs

Tsuda et al. recorded an adherence rate of 66.5% in 88 sessions among 16 hospitalized patients and noted the maintenance of physical performance [56]. The Hoffman et al. study reported a mean adherence rate at the end of Phase I of 96.6% (SD: 3.4%) and of 87.6% (SD: 12.2%) at the end of phase II [52]. To summarize, two studies considered adherence rates as an outcome, but none of the two compared it to standard rehabilitation adherence rates.

In summary, VRR was found to be significantly effective for cancer-related fatigue, cognitive impairment, and CIPN-related balance impairment. VRR was found to be effective for cancer survivors’ pain, but only two studies found it significantly superior to standard rehabilitation. The included studies showed mixed results for the motor impairment outcome, with two studies reporting statistically significant data in favor of VRR and one study reporting statistically significant results in favor of the control group for some of the motor performance outcomes. None of the included studies found a statistically significant effect on lymphedema.

## 4. Discussion

The present review aimed to offer an overview of the present evidence regarding the benefits of the integration of VR for the rehabilitation of the chronic symptoms and impairments of a specific population, cancer survivors. As previously discussed, the impairments and chronic symptoms considered by the present review are indications for and can be treated through rehabilitation programs [15,16,17]. The studies retrieved by our database search found VRR effective on cancer survivors’ pain, accordantly with previous reviews which found VR interventions effective not only for acute but also for chronic pain [57,58,59]. However, only two of the included studies found VRR significantly superior to standard rehabilitation for cancer survivors, so more studies will need to address this comparison. Two of the included studies found statistically significant effects of VR on cancer-related fatigue. This is consistent with the previous literature, which found VRR effective for the treatment of chronic fatigue in other conditions, such as multiple sclerosis [60]. Regarding specifically cancer-related fatigue, however, the previous studies have focused on testing the effects of VR on acute cancer fatigue, for example during procedures such as chemotherapy infusions. Indeed, a 2020 systematic review concluded that VR had a statistically significant beneficial effect on cancer-related fatigue immediately after VR-assisted chemotherapy infusions [61]. Consequently, it must be concluded that more studies are needed to confirm the efficacy of VRR for the long-term treatment of chronic cancer-related fatigue. One study found VRR effective for the treatment of CIPN-related balance impairment, coherently with the results of previous studies on the use of VRR for the treatment of balance impairment secondary to other conditions such as diabetic neuropathy, stroke, and senility [62,63,64]. Two of the included studies considered lymphedema-related excessive arm volume as an outcome, but none found statistically significant evidence in favor of a VRR intervention compared to standard rehabilitation. The included studies also showed mixed results for the motor impairment outcome, with two studies reporting statistically significant data in favor of VRR and one study reporting statistically significant results in favor of the control group for some motor performance outcomes. This result is inconsistent with previous studies showing the efficacy of VRR compared to regular exercise for motor performance and strength outcomes in different conditions, such as cerebral palsy, senility, and after stroke [65,66,67]. One study found VRR effective for the treatment of cognitive impairment in cancer survivors, consistent with the previous literature stating the efficacy of VRR interventions for cognitive impairment [68,69,70,71,72].

Among the included studies, three conducted a home-based intervention [51,52,54]. This area of research is particularly crucial for cancer survivors: as previously discussed, one of the factors contributing to the limited access that cancer patients have to rehabilitative care seems to be represented by the transportation issues resulting from the patients’ disability [16,23,73]. For this reason, many studies have been investigating the potential role of telerehabilitation in improving cancer patients’ access to rehabilitative care [29]. Furthermore, the previous literature has addressed how virtual reality may more generally improve and facilitate remote-assisted and home-based healthcare interventions [26,33,74,75]. Considering more particularly the studies included in our review, Hoffman et al. employed a Wii Fit device to deliver a rehabilitative program of increasing intensity. The program involved only two home visits by a rehabilitation professional, one of which was before the start of the training program to set up the device, later involving only remote phone assistance. The study showed promising results in terms of adherence rates; however, its single-arm design did not allow the authors to conclude whether the VR-implemented program actually improved adherence rates compared to standard facility-based or home-based training programs. Reynolds and colleagues also reported the results of a VRR home-based intervention that did not require assistance from a rehabilitation professional but did not report adherence rates. However, discussing the acceptability of their intervention, they reported a feedback comment which may be found suggestive, although of course far from acceptable as evidence:

“With my lack of mobility that’s resulted from my illness, I really enjoyed the VR as it made me feel like I’m not house bound…”

Feyzioğlu et al., on the other hand, conducted a randomized controlled trial, comparing two home-based interventions, an Xbox 360 Kinect-based intervention and a standard physiotherapy intervention. However, the experimental intervention involved a combination of standard physiotherapy and VRR, as it consisted of a phase of active training through a VRR gaming session and passive mobilization and scar tissue massaging, both performed by the trained physiotherapist. As such, this home-based intervention required the constant physical presence of a rehabilitation professional rather than involving remote assistance. So it must be concluded that more studies are needed to examine whether the VR implementation would facilitate remote supervision and whether the implementation of this technology in home-based interventions would improve the cancer survivors’ adherence. A possible limitation emerging from the overview of the included studies is, however, the compatibility of some applied VRR systems and especially some of their more complex additional devices with home-based interventions in terms of both costs and usability. However, other included studies did test the application of VR devices currently already commercially available, mainly for entertainment and gaming purposes, and which may even be already present in the patients’ houses [48,50,51,54,56]. As previously reported, two of the included trials considered adherence as an outcome [52,56]. However, both consisted of single-arm studies, so more studies are needed to confirm the hypothesis that VRR may actually improve adherence in cancer patients compared to traditional rehabilitation. This result would be consistent with previous studies reporting how VRR may benefit both adherence rates and training intensity [41,42,43,62,76]. More evidence on this subject would be very significant, as many studies highlighted how cancer survivors often discontinue rehabilitation programs as early as within the first 12 months [24]. One of the contributing factors to these statistics seems to be represented by the patient’s lack of confidence and motivation, as standard rehabilitation programs typically require high numbers of repetitions of exercises, which are found to be tiring and boring, when not very frustrating [77]. On this subject, it has been theorized how VRR may increase the patients’ enjoyment and excitement about the rehabilitation task administered, which many researchers argue may benefit both adherence rates and training intensity [41,42,43]. Part of the excitement added by the VR implementation may be explained by the novelty of interacting with a virtual world or even simply wearing an HMD instead of using standard training tools. However, part of its potential in terms of increased engagement seems to derive from the possibility of adding game-like features, rules, and designs to the training tasks, a process named gamification [34,78,79,80]. Indeed, the virtually unlimited possibilities of the virtual scenario design allow adding positive feedback and an exciting narrative to the training activities through the setting of goals, challenges, and competition elements such as score points and badges [79,81,82,83]. In addition, VR scenarios can replicate real-life tasks and situations with the result of greater physical and cognitive fidelity of the trained task to the everyday task the patient needs to reacquire. So, it may be argued that VRR may improve motivation by structuring a more goal-oriented training program compared to the execution of physical exercises in the context of a rehabilitation facility.

Another possible advantage of VRR comes from the multisensorial nature of VR experiences, which allow the stimulation of the patient in a multimodal manner [74]. This is particularly important when it comes to cancer-related disabilities, which, as previously discussed, often derive from the sum of more than one impairment. On this subject, we aim to stress how four of the retrieved studies tested VRR on more than one physical impairment [50,51,53,54]. In addition, three of the included studies considered the effects of VRR on both psychological and physical outcomes [53,54,56], with one also considering cognitive outcomes [53]. Furthermore, we would also like to note how two of the included studies tested VRR systems integrating VR with other technologies [53,55]. In particular, House et al. tested a system consisting of a low-friction robotic rehabilitation table, computerized forearm supports, and a display delivering the non-immersive VR scenario. Schwenk et al. used inertial sensors equipped with gyroscopes and accelerometers on the lower limbs connected to the VRR software, to deliver error-based retraining in the motor tasks required. Many previous studies also integrated VR with other technologies, utilizing the VR software to process the data sent live from different digital rehabilitation tools including treadmills [40,84,85,86,87,88], data gloves [89,90,91], and robotically-assisted orthoses [92,93,94,95,96]. So, regarding this subject, we aim to stress how VR software can represent an integration platform for the function of many devices currently being tested or already clinically used in the rehabilitation field and for cancer survivors. 

## 5. Conclusions

The included studies and the previous literature suggest that VRR may be better tailored to cancer survivors’ needs, such as the need for home-based rehabilitation, the need for incentives for adherence and motivation, and the need for a multimodal approach. More randomized controlled trials are needed to produce evidence on the possible advantages of VRR compared to standard rehabilitative care. In particular, it would be crucial to confirm the hypothesis that VRR may improve adherence rates thanks to its more entertaining nature and multimodal stimulation. Lastly, we wish to encourage the development of new VRR systems and VRR training programs structured to support remote connections in order to allow patients to more easily reach the assistance of healthcare and rehabilitation professionals. Nonetheless, the existence of wide margins for technological development allows us to expect further improvements in the clinical efficacy and usability of VRR systems as well as a reduction in their prices.

## Figures and Tables

**Table 1:** Features of the included studies.

Included Study	Study Design	VRR System	Considered Impairment	Outcome	Conclusions
Atef et al., 2020 [48]	Comparative study	Nintendo Wii games	Post-mastectomy lymphedema	Upper limb function (quickDASH); arm volume	VR training was not inferior to regular proprioceptive neuromuscular facilitation in improving functioning and reducing volume.
Axenie et al., 2020 [49]	Perspective study	Virtual reality avatar-based kinematics assessment and sensorimotor training	Chemotherapy-induced polyneuropathy	Not applicable	Virtual reality software allowed for simultaneous kinematics assessment and multimodal sensorimotor stimulation. In addition, it may facilitate motion training through the use of avatars.
Basha et al., 2021 [50]	Comparative study	Xbox Kinect with games involving upper limb movement	Breast cancer-related lymphedema	Pain (VAS), upper limb function (DASH), shoulder and elbow ROM, hand grip strength, quality of life	VR training was superior to resistance exercises for pain, upper limb function, and shoulder ROM outcomes.
Feyzioğlu et al., 2019 [51]	Comparative study	Xbox Kinect	Post-mastectomy arm and shoulder impairment	Pain (VAS), grip strength, functionality (disabilities of the arm, shoulder, and hand questionnaire), muscle strength, ROM and fear of movement (TKS)	Both standardized therapy and VRR resulted in significant changes in pain, ROM, muscle strength, grip strength, functionality, and TKS scores, without any significant differences between groups. Fear of movement was significantly improved in the VRR group but the standard physiotherapy group displayed more improvement in functionality.
Hoffman et al., 2014 [52]	Randomized non-controlled trial	Nintendo Wii Fit Plus	Post-thoracotomy cancer-related fatigue	Levels of adherence (days of training), exercise performance, cancer-related fatigue (0–10 scale), perceived self-efficacy for fatigue self-management (0–10 scale), perceived self-efficacy for walking 30 min (%)	Non-immersive virtual reality improved both CRF and perceived self-efficacy.
House et al., 2016 [53]	Feasibility study	BrightArm Duo: robotic rehabilitation table, computerized forearm supports, and display	Post-mastectomy arm impairment, depression in cancer survivors	Pain (NRS); arm function (FMA, upper extremity section); bimanual function (CAHAI-9); hand function (JHFT); upper arm autonomy in ADL (UEFI-20); depression (BDI-II); cognitive function (NAB, HVLT-R, BVM-T, TMT);	VR rehabilitation significantly improved 10/11 cognitive parameters and depression scores. In addition, it improved arm function as well.
Reynolds et al., 2022 [54]	Randomized non-controlled trial	Immersive VR headset (Pico Goblin)	Pain, fatigue, depression, anxiety, and stress in metastatic breast cancer patients	Pain (BPI), quality of life (EQ-5D-5L scale), fatigue (FACIT-Fatigue), depression, anxiety, and stress levels, (DASS-SF)	VRR scenarios had significant effects on all considered outcomes. VRR scenarios did not significantly differ in any outcome
Schwenk et al., 2015 [55]	Randomized controlled trial	Non-immersive Virtual Reality software connected to triaxial accelerometers, gyroscopes, and magnetometers	Chemotherapy-induced polyneuropathy	Balance (sway of hip, sway of ankle, center of mass movement), gait speed, fear of falling (FES-I score)	Virtual reality improved balance through patient-tailored, sensor-based exercise but did not improve gait speed and fear of falling
Tsuda et al., 2016 [56]	Randomized non-controlled trial	Nintendo Wii Fit	Physical performance worsening related to chemotherapy and hematological malignancies	Levels of adherence, physical performance (Barthel index), muscle strength, emotive state (hospital anxiety and depression scale)	Virtual reality exercise programs showed good adherence rates (66.5%) and helped maintain physical performance in hospitalized patients.

* Table 1: Features of the included studies. VR: Virtual reality; VAS: visual analogue scale; DASH: disability of the arm, hand, and shoulder questionnaire; ROM: range of motion; TKS: Tampa Kinesiophobia Scale; CRF: cancer-related fatigue; NRS: numeric rating scale; FMA: Fulg-Meyer assessment; CAHAI-9: Chedokee arm and hand activity inventory; JHFT: Jebsen hand function test; ADL: activities of daily living; UEFI-20: upper extremity function index; BDI-II: Beck Depression Inventory, Second Edition; NAB: Neuropsychological Assessment Battery; HVLT-R: Hopkins Verbal Learning Test; BVMT-R: the Brief Visuospatial Memory Test, Revised; TMT: Trail Making Test; FES-I: Falls efficacy scale—international; pain, measured by BPI: (Brief Pain Inventory scale) (BPI); quality of life, measured through the EQ-5D-5L scale; fatigue, measured through the Functional Assessment of Chronic Illness Therapy Fatigue scale (FACIT-Fatigue); and depression, anxiety, and stress levels, measured through the short version of the Depression, Anxiety, and Stress Scales (DASS-SF).

## Data Availability

Not applicable.
